# Risk factors for air leakage during invasive mechanical ventilation in pediatric intensive care units

**DOI:** 10.1186/s40001-022-00858-x

**Published:** 2022-10-28

**Authors:** Ling Ma, Miao Yin, Xi-Lun Yang, Wei Xu

**Affiliations:** 1 Department of Anesthesiology, Shengjing Hospital of China Medical University, No. 36 Sanhao Street, Heping District, Shenyang, 110004 Liaoning China; 2 Department of Pediatrics, Shengjing Hospital of China Medical University, No. 36 Sanhao Street, Heping District, Shenyang, 110004 Liaoning China

**Keywords:** Invasive mechanical ventilation, Air leak, Pneumothorax, Subcutaneous emphysema, Pneumomediastinum, Children

## Abstract

**Purpose:**

This study aimed to investigate air leakage during invasive mechanical ventilation (IMV) in a pediatric intensive care unit (PICU) and explore potential risk factors.

**Methods:**

We conducted a retrospective cohort study of children who underwent IMV in a single-center PICU in a tertiary referral hospital. Air leakage risk factors and factors associated with an improved outcome were assessed.

**Results:**

A total of 548 children who underwent IMV were enrolled in this study. Air leakage occurred in 7.5% (41/548) of the cases in the PICU. Air leakage increased the duration of IMV and hospitalization time. Multivariate logistic regression analysis showed a higher risk of air leakage during IMV for PICU patients with acute respiratory dyspnea syndrome (ARDS) (OR = 4.38), a higher pediatric critical illness score (PCIS) (OR = 1.08), or a higher peak inspiratory pressure (PIP) (OR = 1.08), whereas the risk was lower for patients with central respiratory failure (OR = 0.14). The logistic model had excellent predictive power for air leakage, with an area under the curve of 0.883 and tenfold cross-validation. Patients aged between 1 and 6 years who were diagnosed with measles or pneumonia and had a low positive end-expiratory pressure (PEEP) or high PaO_2_/FiO_2_ ratio were associated with improved outcomes. Patients diagnosed with central respiratory failure or congenital heart diseases were associated with less desirable outcomes.

**Conclusions:**

Patients with ARDS, a higher PCIS at admission or a higher PIP were at higher risk of air leakage.

## What is known

- Air leakage is a serious hazard during IMV and can increase ICU stay and mortality in patients
- To date, studies on IMV-related air leakage in pediatric patients are insufficient.

## What is new

- This study showed higher risks of air leakage during IMV for PICU patients with ARDS, a higher PCIS, or a higher PIP.
- This study showed that patients aged between 1 and 6 years, those diagnosed with measles or pneumonia, and those with a low PEEP or high PaO_2_/FiO_2_ ratio were associated with improved outcomes.

## Introduction

Air leakage is defined as the leakage of air from an air-containing cavity into spaces that, under normal circumstances, do not contain air [1]; this includes pneumothorax, pneumomediastinum, pneumopericardium, pneumoperitoneum, and subcutaneous emphysema [2]. Air leakage can be spontaneous or secondary to pulmonary inflammatory disease. Air leakage is rare, but can result in life-threatening complications. The incidence of air leakage has been reported to be approximately 3.4/100,000 in children among the normal population [3], and pulmonary air leakage occurs in 4.8% of children in the pediatric intensive care unit (PICU) [4]. Studies have shown that air leakage can increase ICU stay and mortality in patients [5, 6]. Mechanical ventilation, as an important means of life support for patients with respiratory failure, can also cause various types of injuries [7]. Pulmonary air leakages present a serious hazard for patients on mechanical ventilation and are the main type of iatrogenic air leakage [8]. A prolonged, repeated, and forced supply of high pressure and a large volume of gas into the lungs during the invasive mechanical ventilation (IMV) process can induce damage to the alveolar and small airway structures, pulmonary interstitial edema, inflammatory factor accumulation, and reduced pulmonary surfactant formation, which can lead to rupture of the alveolar wall [9, 10]. When entering the chest, pericardium, subcutaneous tissue, and perimediastinum, gases can cause increased chest pressure, lung atrophy, venous return obstruction, cardiac output reduction, and lung ventilation dysfunction, manifested as varying degrees of dyspnea, hypotension, and other clinical abnormalities. In severe cases, these conditions can quickly endanger patients’ lives [11]. Therefore, effective prevention, early diagnosis, and timely treatment are extremely important for the reduction of air leakage incidence and mortality.

Only a few clinical studies have studied air leakage, a complication of invasive mechanical ventilation in children. As the largest pediatric intensive care center in Northeast China, our center has a large number of patients with respiratory failure. There was also a small regional outbreak of childhood measles throughout the study period.

Our primary objective was to determine the risk factors for air leakage during IMV in ventilated patients admitted to the PICU at Shengjing Hospital of China Medical University over a 5-year period. The secondary objective was to identify factors to improve outcomes in patients with air leakage.

## Materials and methods

### Patient selection

This retrospective study was conducted in a 35-bed PICU at the Shengjing Hospital of China Medical University, a tertiary referral hospital. The Medical Ethics Committee of Shengjing Hospital of China Medical University approved the study protocol (Number 2022PS732K). This was approved with a nonsignificant risk determination, which led to the waiver of informed consent.

#### Inclusion criteria

This study included all children under IMV for more than 12 h who were aged between 28 days and 14 years old and were admitted to the PICU of Shengjing Hospital between January 1, 2014, and December 31, 2018. There were 588 patients in total.

#### Exclusion criteria

Patients were excluded under the following conditions: (1) children who underwent elective thoracic surgery or procedures, such as thoracentesis; (2) children with the following malformations of the airway or lung: bronchiectasis, pulmonary cyst, bullae of the lung, idiopathic pulmonary fibrosis, and bronchopulmonary dysplasia; (3) children who had air leakage before IMV; and (4) children with incomplete data.

### Data collection

An air leakage diagnosis was based on the following two criteria: (1) evidence of air leakage on chest CT and (2) gas discharge after an emphysema incision or thoracentesis. The following data were collected from all participants: (1) general information for the child, including their age, sex, weight, date of admission, pediatric critical illness score (PCIS) [12] upon admission to the PICU, and length of hospital stay; (2) comorbidities and causes of ventilator support, including the degree of pneumonia, asthma, ARDS, CHD, CNS disease, and postoperation; (3) diseases occurring during IMV, including ventilator-related pneumonia, ARDS, multiple organ dysfunction syndrome (MODS), and sepsis; (4) latest respiratory parameters before air leakage occurrence, including the mode, peak inspiratory pressure (PIP), positive end-expiratory pressure (PEEP), respiratory rate (RR), fraction of inspiration O_2_(FiO_2_), partial pressure of oxygen in arterial blood (PaO_2_), PaO_2_/FiO_2_ ratio, and mechanical ventilation time; and (5) treatment processes, including cardiopulmonary resuscitation, high-frequency ventilation, and the time of thoracic puncture or closed-tube thoracostomy.

Data were collected from electronic medical records (EMRs) by trained investigators who were independent of the clinical teams. Daily mechanical ventilation data were collected during the first 7 days of IMV. Laboratory data were collected during the first 3 days of the PICU stay. An extensive round of data cleaning was performed before the analysis to check for data accuracy and outliers.

### Respiratory management

Ventilators (Evita 2 Dura, Dräger, Germany) in pressure control ventilation mode with a tidal volume set at 6–8 mL/kg were used for IMV patients. Sufentanil for analgesia was used at the start of mechanical ventilation. The sufentanil loading and maintenance doses were 0.3–0.5 µg/kg and 0.03–0.05 µg/kg/h, respectively. All patients underwent midazolam sedation. The midazolam loading and maintenance doses were 0.1–0.3 mg/kg and 2–8 µg/kg/h, respectively. When conventional mechanical ventilation failed, a trial of high-frequency oscillatory ventilation (HFOV) was considered. Ramsay scores were kept at 3–5.

Neuromuscular blocking agents were used to reduce patient-to-ventilator asynchronies and to help patients better adapt to protective ventilation. Rocuronium at 5–10 µg/kg/min was used in our PICU.

### Criteria for disease outcomes

Disease outcomes were defined as follows: (1) recovery: clinical symptoms and signs had improved; chest X-ray or CT showed a reduction or disappearance in the air leakage; (2) discharge against medical suggestions: clinical symptoms, signs, and chest X-ray or chest CT findings had not improved, but the family ended the treatment in the PICU; and (3) death in the ICU: effective treatment included improvement and ineffective treatment included discharge against medical suggestions and in-hospital death.

### Statistical analysis

The clinical characteristics and outcomes of mechanically ventilated patients who developed air leakage (cases) were compared with those who did not develop air leakage (controls).

R (version 4.1.2) was used for data processing and analysis. Measurement data were expressed as the median and interquartile range (IQR) [median (IQR)]. The Mann–Whitney *U* test was used for comparisons between groups. Count data were expressed as counts and percentages, and comparisons between groups were performed using the *χ*^2^ test or Fisher’s exact test.

We used linear regression to assess the association between air leakage and length of hospital stay and duration of ventilation. Due to the skewed distribution, we used the log-transformed duration data in the regression. In the model, we also considered age, sex, ARDS, asthma, measles, pneumonia, postoperative, central respiratory failure, CHD, PCIS, PIP, PEEP, PaO_2_/FiO_2_ ratio, and type of air leakage as potential covariates and used a stepwise procedure for variable selection. Logistic regression analysis with the stepwise procedure for variable selection was used to assess the association between air leakage and the following variables: age, sex, ARDS, asthma, measles, pneumonia, postoperative, central respiratory failure, CHD, PCIS, PIP, PEEP, and PaO_2_/FiO_2_ ratio. The same method was also used to evaluate the factors associated with an improved outcome versus the less desirable outcomes of death or discharge against medical suggestion. P < 0.05 was considered the threshold for statistical significance. We used the “vfold_cv” function in the *tidymodels* R package to calculate the area under the curve (AUC) of the logistic models with tenfold cross-validation.

## Results

During the study, 588 patients were admitted to the PICU. Forty patients were excluded based on the exclusion criteria, leaving 548 patients eligible for this study. Among them, 41 patients (7.5%) experienced air leakage.

### Clinical features

In this study, the median age of the patients was 1 year (y), with a maximum age of 14 y and a minimum age of 2 months. We categorized the patients into three age groups: infant (≤ 1 y), preschool (> 1 y and ≤ 6 y), and school-age (> 6 y). A total of 300 patients (54.7%) were 1 y old or younger, 141 patients (25.7%) were between 1 y old and 6 y old, and 107 patients (19.5%) were older than 6 y. No association between age and air leakage was observed (Table 1).

**Table 1:** Demographic and clinical characteristics of the two groups

	Air leakage (*n* = 41)	No air leakage (*n* = 507)	Total (*n* = 548)	*p*
Categorical variables^a^				
Age				1
≤ 1y	22 (0.54)	278 (0.55)	300 (0.55)	
1–6 y	12 (0.29)	129 (0.25)	141 (0.26)	
7–14 y	7 (0.17)	100 (0.2)	107 (0.2)	
Sex				
Male	27 (0.66)	308 (0.61)	335 (0.61)	0.62
Female	14 (0.34)	198 (0.39)	212 (0.39)	
ARDS	22 (0.54)	40 (0.08)	62 (0.11)	< 0.001
Asthma	2 (0.05)	17 (0.03)	19 (0.03)	0.65
Measles	5 (0.12)	8 (0.02)	13 (0.02)	0.002
CHD	7 (0.17)	97 (0.19)	104 (0.19)	0.84
Postoperative	5 (0.12)	41 (0.08)	46 (0.08)	0.37
Central respiratory failure	2 (0.05)	199 (0.39)	201 (0.37)	< 0.001
HFOV	14 (0.34)	13 (0.03)	27 (0.05)	< 0.001
Pneumonia	33 (0.8)	370 (0.73)	403 (0.73)	0.36
Outcome				
Recovery	20 (0.49)	278 (0.55)	298 (0.54)	0.42
Death in the ICU	9 (0.22)	55 (0.11)	64 (0.12)	
Discharge	12 (0.29)	175 (0.34)	187 (0.34)	
Subcutaneous emphysema	21 (0.51)			
Pneumothorax	34 (0.83)			
Pneumomediastinum	16 (0.39)			
Continuous variables^b^				
Hospital stay (days)	18 (20)	13 (16)	14 (17)	< 0.001
PCIS	98 (8)	96 (10)	96 (10)	0.007
PIP	32 (12)	22 (6)	24 (8)	< 0.001
PEEP	8 (6)	3 (4)	3.5 (4)	< 0.001
RR	30 (10)	25 (8)	30 (5)	< 0.001
PaO2	89 (81)	123 (59.5)	122.5 (62.2)	0.007
FiO2	0.5 (0.3)	0.3 (0.2)	0.4 (0.2)	< 0.001
PaO2/FiO2 ratio	190 (182.8)	338.1 (230)	330 (232.6)	< 0.001
IMV duration (hours)	240 (288)	120 (144)	144 (168)	< 0.001
Leak since ventilation (hours)	120 (120)			

*ARDS* acute respiratory dyspnea syndrome; *CHD* congenital heart disease; *HFOV* high-frequency oscillatory ventilation; *NMBA* neuromuscular blocking agent; *PCIS* pediatric critical illness score; *PIP* peak inspiratory pressure; *PEEP* positive end-expiratory pressure, *RR* respiratory rate, *PaO*_*2*_ partial pressure of oxygen in arterial blood; *FiO*_*2*_ fraction of inspiration oxygen

*P value*s of the Mann‒Whitney *U* test are shown

^a^Counts and proportions are shown for categorical variables. *P value*s of Fisher’s exact test are shown

^b^The median and interquartile range are shown for continuous variables

Among the 41 patients with air leakages, 27 were male (66%), and 14 were female (34%). Similar proportions of male and female patients were present in the groups with and without air leakages (Table 1).

Air leakages occurred within 1 hour (h) to 20 days (d) after IMV. The median time was 5 d. A total of 9 patients (22%) experienced air leakage within 24 h after IMV, 23 patients (56%) experienced air leakage between 1 and 7 d, and 9 patients (22%) experienced air leakage at more than 7 d.

**Fig. 1:**
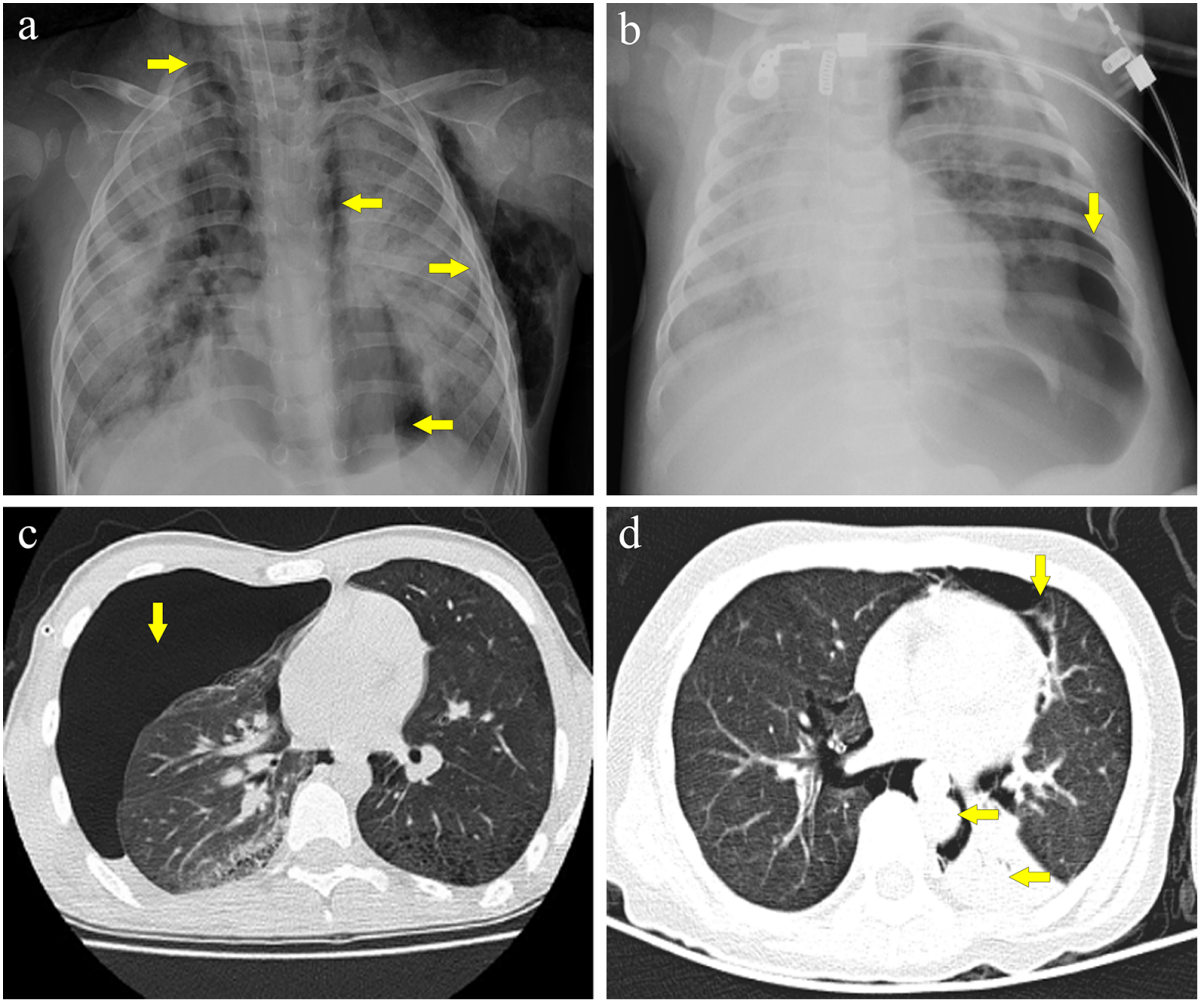
Imaging presentation. **a** Anterior–posterior chest radiograph of a 2-year-old patient showing subcutaneous emphysema and pneumomediastinum. **b** Anterior–posterior chest radiograph of a 3-month-old patient showing a left pneumothorax. **c** High-resolution computed tomography scan of a 3-year-old patient showing a right pneumothorax. **d** High-resolution computed tomography scan of a 10-year-old patient showing pneumomediastinum and pneumothorax. The right arrow indicates subcutaneous emphysema, the downward arrow indicates pneumothorax, and the left arrow indicates pneumomediastinum

Pneumothorax was right-sided in 26 patients (63.4%) and left-sided in 15 patients (36.6%). Seven patients (17.1%) had bilateral pneumothoraces. Pneumomediastinum occurred in 16 patients (39.0%). Subcutaneous emphysema occurred in 21 patients (51.2%). Among the patients diagnosed with subcutaneous emphysema, 8 patients (38.1%) were complicated with both pneumomediastinum and pneumothorax, 7 patients (33.3%) were diagnosed with concurrent pneumothorax only, and 5 patients (23.8%) were diagnosed with concurrent pneumomediastinum only. Pneumomediastinum was diagnosed independently of subcutaneous emphysema and pneumothorax in only one patient (2.4%) (Table 1). The typical radiological manifestations are shown in Fig. 1.

The median duration of mechanical ventilation and length of hospitalization of patients with and without air leakage were 10 d vs. 5 d and 18 d vs. 13 d, respectively (*P* < 0.001). The comparisons of mechanical ventilation duration and length of hospitalization stay between the air leakage group and no air leakage group were statistically significant (Table 1).

We used multiple linear regression to assess the association between air leakage and the duration of ventilation and length of hospital stay while adjusting for potential confounders and observed that air leakage was associated with a 69% (95% CI 27–126%, *P* < 0.001) increase in ventilation duration and an 83% (95% CI 33–151%, *P* < 0.001) increase in length of hospital stay.

### Air leakage risk factors

Univariate analysis identified the following variables as being significantly associated with air leakage: ARDS, measles, CNS, HFOV, hospital stay, PCISs, PIP, PEEP, RR, PaO_2_, FiO_2_, PaO_2_/FiO_2_ ratio, and ventilation duration (Table 1).

**Fig. 2:**
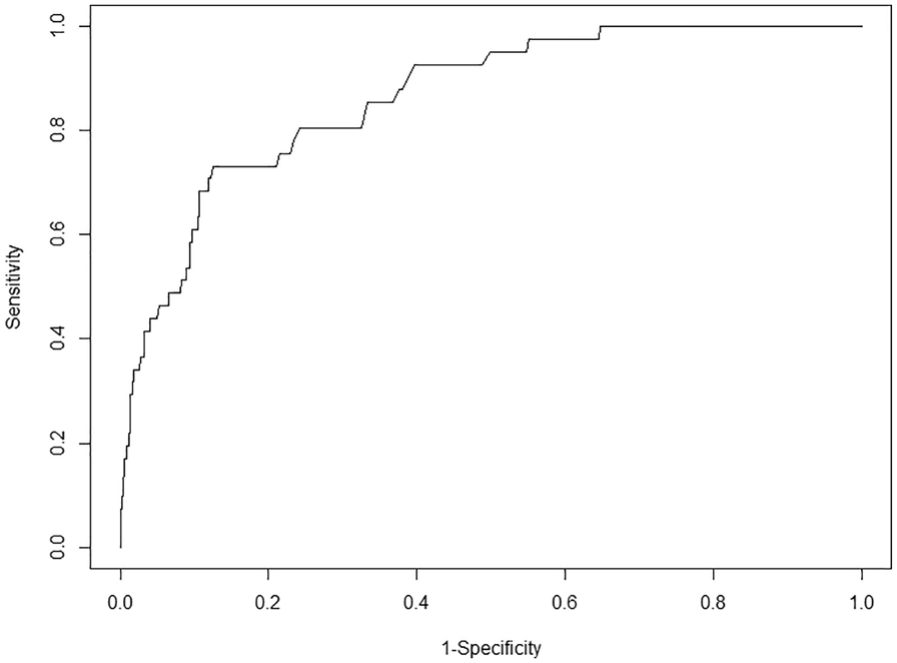
Receiver operating characteristic (ROC) curve of the logistic model for air leakage

Logistic regression was used to assess air leakage risk factors and included age, sex, ARDS, asthma, measles, pneumonia, CHD, postoperative, central respiratory failure, PCIS, PIP, PEEP, and PaO_2_/FiO_2_ ratio as the potential predictors in the model. After the stepwise procedure for variable selection, four variables remained significant in the final model, as shown in Table 2. A higher risk was observed in patients with ARDS (OR = 4.38, 95% CI 1.88, 10.2), a higher PCIS (OR = 1.08, 95% CI 1.03, 1.13), and a higher PIP (OR = 1.08, 95% CI 1.03, 1.14). A lower risk was observed in patients with central respiratory failure (OR = 0.14, 95% CI 0.03, 0.62). This model had excellent predictive power with an AUC of 0.863 and tenfold cross-validation (Fig. 2).

**Table 2:** Logistic regression of factors associated with air leakage

Variable	OR	95% CI	*P* value
ARDS	4.38	(1.88, 10.2)	< 0.001
CNS diseases	0.14	(0.03, 0.62)	0.01
PCIS	1.08	(1.03, 1.13)	0.003
PIP	1.08	(1.03, 1.14)	0.002

*ARDS* acute respiratory dyspnea syndrome; *CNS* central nervous system; *PCIS* pediatric critical illness score; *PIP* peak inspiratory pressure

### Factors associated with patient recovery

In the stepwise logistic regression to evaluate factors associated with improved patient outcomes, the following variables were considered: age, sex, air leakage, ARDS, asthma, measles, pneumonia, CHD, postoperative, central respiratory failure, PCIS, PIP, PEEP, PaO_2_/FiO_2_ ratio and air leak type_._ Air leakages were not associated with the treatment outcomes and did not remain in the final model. However, an age > 1 and ≤ 6 (OR = 1.76, 95% CI 1.1, 2.82), a diagnosis of measles or pneumonia (OR = 9.15, 95% CI 1.89, 44.3; OR = 2.17, 95% CI 1.4, 3.34), and the PaO_2_/FiO_2_ ratio (OR = 1.001, 95% CI 1.0001, 1.003) were associated with an increased chance of patient recovery in the final model. A diagnosis of a CNS disease (OR = 0.67, 95% CI 0.43, 1.03), a diagnosis of a CHD (OR = 0.36, 95% CI 0.21, 0.6), and an increased value of PEEP (OR = 0.84, 95% CI 0.78, 0.9) were associated with a decreased probability of an improved patient outcome (Table 3).

**Table 3:** Logistic regression of factors associated with an improved patient outcome

Variable	OR	95% CI	*p*
Age (1–6 y)	1.76	(1.10, 2.82)	0.04
Measles	9.15	(1.89, 44.32)	0.006
Pneumonia	2.17	(1.40, 3.34)	< 0.001
PaO_2_/FiO_2_	1.001	(1.0001,1.003)	0.05
CNS diseases	0.67	(0.43, 1.03)	0.067
CHD	0.36	(0.21, 0.60)	< 0.001
PEEP	0.84	(0.78, 0.90)	< 0.001

*PaO*_*2*_ partial pressure of oxygen in arterial blood; *FiO*_*2*_ fraction of inspiration oxygen *CNS*: central nervous system; *CHD* congenital heart disease; *PEEP* positive end-expiratory pressure

## Discussion

This retrospective study focused on pediatric patients undergoing mechanical ventilation, analyzing the associated risk factors for air leakage and the clinical implications with respect to their outcomes. In this single-center study, the percentage of air leakages was 7.5% in PICU patients under IMV. Patients with ARDS, noncentral respiratory failure, an increased PCIS, or a higher PIP were more likely to experience an air leakage. During IMV, the occurrence of air leakages can increase a patient's IMV duration and length of hospitalization stay.

According to the literature, depending on the severity of the disease, treatment, and ventilator management, the incidence of ventilator-related air leakages varies from 3 to 15% [13, 14]. The total percentage of IMV-related air leakages in the study hospital in the past 5 years was 7.5%, which is consistent with the literature. In addition, this study concluded that air leakages increased the length of hospitalization stay and the duration of mechanical ventilation in patients, in agreement with previous reports of adult patients [15, 16].

Furthermore, subcutaneous emphysema is mostly a complication of pneumothorax [17]. In this study, 20 cases of subcutaneous emphysema were associated with pneumothorax or pneumomediastinum. However, we did not observe any significant difference in ventilation duration or length of hospital stay across different air leakage subtypes.

Yadav et al. [18] reported that pediatric ARDS resulted in a significant burden to a developing country’s PICU and was associated with significantly higher mortality. Pneumothorax occurred in 30% of invasively ventilated children. Eisner et al. [19] studied air leakages using a cohort of 718 patients with ARDS and reported 93 patients (13%) with barotrauma. In the first 4 days of mechanical ventilation, the cumulative incidence rate of air leakages more than doubled, from 5.8% on the first day to 13% on the fourth day. The median time of air leakage in this study was 5 days, indicating that mechanical ventilation should be closely monitored during the first week when air leakage is most likely to occur.

Literature on the pathophysiology during pneumothorax is outrageously scarce compared with its high incidence [20]. An experimental study in rats showed that PIP is the only relevant factor that determines pneumothorax [21]. Briassoulis et al. showed that mechanical ventilation produced air leakages, particularly when high peak airway pressures (barotrauma) or large tidal volumes (volutrauma) were delivered by the ventilator [7]. Most cases of pneumothorax are iatrogenic and caused by barotrauma [22, 23]. This study confirmed that a higher PIP was associated with air leakage, although lung protective strategies had already been performed during the study period.

A previous study showed that a low PCIS predicted an unfavorable prognosis of gastric perforation beyond the neonatal period in 20 pediatric patients [24]. In a retrospective analysis of 45 children with ARDS and hematological neoplasms, the PCIS and average fluid volume in the first 3 days were independent risk factors for predicting death [25]. The lower the PCIS was, the more serious the organ damage. However, in this study, an association between a higher PCIS and air leakages was observed. This paradoxical association may be because the PCIS is a comprehensive score based on the injury of multiple organs and systems. It is determined not only by pulmonary conditions, but also by diseases of other vital organs, such as the circulation, liver, kidney, and coagulation. In this case, the incidence of air leakage is not high when other organs outside the lung are seriously injured. However, if the PCIS is high, it means that other organs are not seriously injured. Most of the time, the reason for IMV in these patients is a problem with the lung itself, which may be more prone to air leakage. Therefore, a patient may have a higher PCIS but still have poor pulmonary function and therefore be at higher risk of air leakage.

For children undergoing constant-frequency mechanical ventilation, a series of studies have suggested that HFOV can reduce the average airway pressure and avoid oxygen leakage while improving oxygenation [26, 27]. Several studies have suggested that HFOV has a similar or higher incidence rate of air leakage than normal frequency ventilation [28]. Bateman ST et al. showed that early HFOV was associated with a longer duration of mechanical ventilation [29]. In the PICU study, HFOV was used as a rescue therapy in cases of failure of the conventional ventilation mode to provide good oxygenation in severe ARDS patients. The use of HFOV is often combined with the use of continuous nitrogen monoxide inhalation, even for extracorporeal membrane oxygenation. The study data showed that HFOV was not an independent risk factor for IMV air leakage. Multiple factors beyond ventilation can contribute to children’s outcomes and controlling for these variables is extremely challenging in a retrospective analysis of this size. Therefore, we cannot confirm the superiority of HFOV to the conventional ventilation mode, which is consistent with a previous study [29].

The study data showed that central respiratory failure was associated with a low risk of air leakage, but poor outcomes were observed in patients undergoing IMV in the PICU. The diagnosis of CHD was also associated with a poor outcome. Extrapulmonary diseases, mostly CNS and CHD, usually with mild-to-moderate pulmonary inflammation and injury, tend to have better ventilator parameters and less trigger pressure, hence the lower risk of air leakage. It is hypothesized that preexisting severe diseases, along with IMV use and the inappropriate use of PEEP, may have been responsible for the poor outcomes in the studied patients. Therefore, it is more important to provide timely and effective adjunctive treatment to protect the function of vital organs of patients with central respiratory failure or CHD.

Recently, there has been a resurgence of measles in several countries [30]. In 2014, a measles pandemic was reported in the northeast part of China. In pediatric patients, measles remains a serious threat. Spontaneous air leakages were reported in 0.5 to 2.4% of hospitalized children in the 1990s [31]. In the PICU study, measles infections were shown in 5/41 (12.2%) patients with air leakage and 8/507 (1.6%) patients without air leakage, with a total occurrence of 13/548 (2.4%) IMV patients experiencing air leakage, similar to previous reports. Fortunately, patients diagnosed with measles tended to have better outcomes in the PICU study, as did patients diagnosed with pneumonia.

Kagan et al. conducted a retrospective study in a tertiary center in Israel and reported that pneumothorax secondary to ARDS was associated with a worse outcome, while other etiologies, mainly bacterial pneumonia, were associated with a favorable outcome [4]. In this study, patients in the age range of 1 y to 6 y old with a diagnosis of measles or pneumonia and with a higher PaO_2_/FiO_2_ ratio had an increased chance of an improved outcome.

A major strength of this study is that the cohort was based on a relatively large group of children with all-cause air leakage. Another strength of this study is the PICU setting, which supplied accurate data through an EMR system. However, this study has several limitations. The primary limitation of this study is its retrospective design and that the associations identified cannot establish causation. Second, this study was conducted in a single center, hence limiting its external validity. Later, larger studies at multiple institutions need to be conducted to draw more substantial scientific conclusions. Third, although the PCIS is widely used in China and several studies have verified its comparability with other worldwide scoring systems, such as the PRISM III and PELOD-2, due to our EMR limitation, commonly used risk mortality scores could not be obtained.

## Conclusions

In conclusion, this study found that the main risk factors for air leakage during IMV in the PICU are a high PIP, ARDS, and a high PCIS. Air leakages are less likely to occur in patients in the study PICU who are diagnosed with central respiratory failure.

## Data Availability

Wei Xu is the guarantor of this work and had full access to all the data in the study. Data will be made available upon request.

## Abbreviations

AUC: Area under the curve
ARDS: Acute respiratory distress syndrome
CHD: Congenital heart disease
CNS: Central nervous system
EMR: Electronic medical record
FiO_2_: Fraction of inspired oxygen
HFOV: High-frequency oscillatory ventilation
IMV: Invasive mechanical ventilation
IQR: Interquartile range
MODS: Multiple organ dysfunction syndrome
PaO_2_: Partial pressure of oxygen in arterial blood
PCIS: Pediatric critical illness score
PEEP: Positive end-expiratory pressure
PICU: Pediatric intensive care unit
PIP: Peak inspiratory pressure
ROC: Receiver operating characteristic
RR: Respiratory rate
